# Netazepide, an Antagonist of Cholecystokinin Type 2 Receptor, Prevents Vincristine-Induced Sensory Neuropathy in Mice

**DOI:** 10.3390/ph17020144

**Published:** 2024-01-23

**Authors:** Amandine Bernard, Mohamad Mroué, Sylvie Bourthoumieu, Malcolm Boyce, Laurence Richard, Franck Sturtz, Claire Demiot, Aurore Danigo

**Affiliations:** 1 NeurIT Neuropathies et Innovations Thérapeutiques UR 20218, Faculties of Medicine and Pharmacy, University of Limoges, 87025 Limoges, France; amandine.bernard@unilim.fr (A.B.); mohamad.mroue@unilim.fr (M.M.); sylvie.bourthoumieu@unilim.fr (S.B.); laurence.richard@unilim.fr (L.R.); franck.sturtz@unilim.fr (F.S.); claire.demiot@unilim.fr (C.D.); 2 Department of Cytogenetic, Medical Genetic and Reproduction Biology, University Hospital of Limoges, 87042 Limoges, France; 3 Hammersmith Medicines Research Limited and Trio Medicines Limited, 44 Cumberland Avenue, London NW10 7EW, UK; mboyce@hmrlondon.com; 4 Department of Neurology, Reference Center for Rare Peripheral Neuropathies, University Hospital of Limoges, 87042 Limoges, France; 5 Department of Biochemistry and Molecular Genetics, University Hospital of Limoges, 87042 Limoges, France; 6 Transversal and Territorial Therapeutic Education Unit (UTTEP87), University Hospital of Limoges, 87042 Limoges, France

**Keywords:** chemotherapy-induced peripheral neuropathy, vincristine, allodynia, netazepide, CCK2R

## Abstract

Among the vinca-alkaloid class, vincristine is a potent chemotherapeutic agent with significant neurotoxic effects and is employed to address a wide spectrum of cancer types. Recently, the therapeutic potential of the cholecystokinin type 2 receptor (CCK2R) as a target for vincristine-induced peripheral neuropathy (VIPN) was demonstrated. In this study, the impact of preventive CCK2R blockade using netazepide (Trio Medicines Ltd., London, UK) was investigated in a mouse model of vincristine-induced peripheral neuropathy. Netazepide is a highly selective CCK2R antagonist under development for the treatment of patients with gastric neuroendocrine tumors caused by hypergastrinemia secondary to chronic autoimmune atrophic gastritis. Vincristine-induced peripheral neuropathy was induced by intraperitoneal injections of vincristine at 100 µg/kg/d for 7 days (D0 to D7). Netazepide (2 mg/kg/d or 5 mg/kg/d, per os) was administered one day before vincristine treatment until D7. Vincristine induced a high tactile allodynia from D1 to D7. VIPN was characterized by dorsal root ganglion neuron (DRG) and intraepidermal nerve fiber (IENF) loss, and enlargement and loss of myelinated axons in the sciatic nerve. Netazepide completely prevented the painful symptoms and nerve injuries induced by vincristine. In conclusion, the fact that netazepide protected against vincristine-induced peripheral neuropathy in a mouse model strongly supports the assessment of its therapeutic potential in patients receiving such chemotherapy.

## 1. Introduction

Chemotherapy-induced peripheral neuropathy (CIPN) is characterized by sensory and/or motor dysfunction, according to the anticancer drug administered. Vincristine is a commonly used and effective chemotherapeutic drug for the treatment of a wide range of cancer types, especially in children and young adults with lymphoma and acute lymphoblastic leukemia [1]. Vincristine is the most neurotoxic of the vinca alkaloid family and can lead to the onset of vincristine-induced peripheral neuropathy [2]. Other main adverse drug effects (ADR) of vincristine include alopecia, myelosuppression, and gastrointestinal toxicity [3]. Vincristine-induced peripheral neuropathy is the side effect experienced by almost all children who receive vincristine [4,5,6,7,8]. Those patients develop neuropathic pain such as tactile allodynia and hyperalgesia [9]. Vincristine binds to free tubulin in the cytoplasm, causing it to aggregate into non-functional polymers. Free tubulin is then unavailable for incorporation into microtubules [10], and cancer cells are blocked in metaphase. This phenomenon may be implied in the onset of peripheral neuropathy because of a disorganization of the axonal microtubule network [11]. However, other mechanisms occurred, such as neuronal hyperexcitability, neuroinflammation, and oxidative stress [8]. Duloxetine, the only recommended drug, alleviates chemotherapy-induced painful symptoms in adult patients. However, in children, the only known method of treatment is dose reduction, the delay or discontinuation of the neurotoxic molecule, which reduces the patient’s potential for survival. It is noteworthy that some non-pharmacological interventions showed promising effects on sensory symptoms and on quality of life, such as physical exercise and acupuncture [12,13,14]. None of the preventive therapies tested so far have shown significant clinical efficacy, probably because the exact pathophysiological mechanism of vincristine-induced neuropathic pain remains unclear [15,16]. Thus, there is an important unmet need for an effective preventative treatment. Several studies have shown a strong link between the cholecystokinin type 2 receptor (CCK2R) and the nociceptive process [17,18]. CCK2R belongs to the CCKergic system, which also includes the CCK neuropeptide and the CCK1R receptor [19,20]. The CCK2R is found mainly in the gastric enterochromaffin-like (ECL) cells in the stomach and in regions of the brain associated with pain modulation, as well as other functions including memory, anxiety, and thermoregulation. The few studies focused on the expression of CCK2R in the peripheral nervous system have shown that CCK2R RNA is expressed in the dorsal root ganglion (DRG) in physiological conditions and overexpressed in traumatic nerve injuries [21,22,23]. Previous preclinical studies have highlighted the potential for the blockade of CCK2R in the management of pain [23,24,25]. Thus, we believe that CCK2R could be a therapeutic target for CIPN in patients. Recently, we demonstrated overexpression of the cck2r gene in DRG in a mouse model of vincristine-induced peripheral neuropathy [26]. In that model, preventive treatment with the CCK2R antagonists, proglumide (a non-specific CCK2R antagonist) or Ly225910 (a selective CCK2R antagonist), prevented the painful symptoms of vincristine-induced peripheral neuropathy, but only Ly225910 alleviated the nerve injuries induced by the anticancer agent. Also, blockade of CCK2R showed analgesic and neuroprotective effects. Several other CCK2R antagonists, including YF476 (netazepide) [27], have been described [28]. Netazepide has been called the gold standard for gastrin/CCK2R antagonists [29] and has been the subject of several clinical pharmacology trials [30,31,32,33,34] and trials for the treatment of patients with gastric neuroendocrine tumors caused by hypergastrinaemia secondary to chronic autoimmune atrophic gastritis [35] and for other conditions associated with hypergastrinaemia. In this study, our goal was to evaluate the effect of netazepide on pain behavior and nerve injuries induced by vincristine in a mouse model.

## 2. Results

### 2.1. Netazepide at 2 and 5 mg/kg Prevents Vincristine-Induced Allodynia

**Figure 1:**
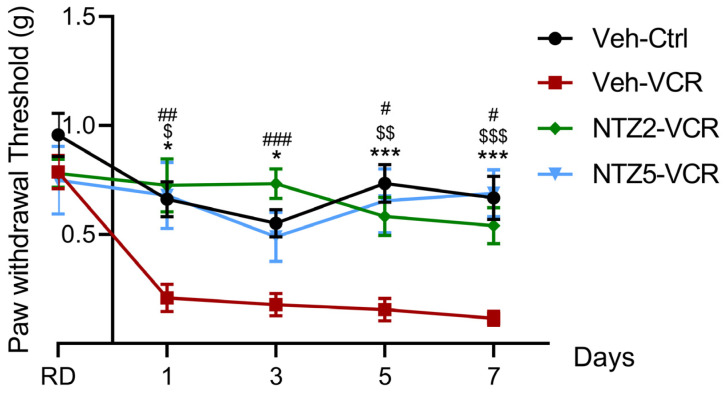
Effects of netazepide (2 or 5 mg/kg) on mechanical allodynia induced by vincristine in mice. Mechanical sensitivity was evaluated using the von Frey filament test at D1, D3, D5, and D7. n = 10 mice per group, * *p* < 0.05, *** *p* < 0.001, Veh-VCR vs. Veh-Ctrl. $ *p* < 0.05, $$ *p* < 0.01, $$$ *p* < 0.001, Veh-VCR vs. NTZ5-VCR, # *p* < 0.05, ## *p* < 0.01, ### *p* < 0.001 Veh-VCR vs. NTZ2-VCR, RD: reference day, Ctrl: control; NTZ2: netazepide at 2 mg/kg; NTZ5: netazepide at 5 mg/kg; Veh: vehicle; VCR: vincristine.

Netazepide and its vehicle (50% PEG 300, 40% NaCl 0.09%, 10% DMSO) had no significant effect on mechanical responses in the Ctrl groups on D1, D3, D5, or D7. As already described [36], mice of the Veh-VCR group developed significant mechanical allodynia from D1 to D7 compared with mice of the Veh-Ctrl group (D1: *p* = 0.0171; D3: *p* = 0.0279; D5: *p* = 0.0001; D7: *p* = 0.0003). Vincristine-administered mice treated with netazepide did not develop mechanical allodynia. Indeed, there was no significant difference between NTZ5-VCR and Veh-Ctrl mice from D1 to D7 (D1: *p* = 0.9991, D3: *p* = 0.9657, D5: *p* = 0.9332, D7: *p* = 0.9984) (Figure 1). Likewise, there was no significant difference between NTZ2-VCR and Veh-Ctrl mice for mechanical allodynia (D1: *p* = 0.9646, D3: *p* = 0.5221, D5: *p* = 0.6714, D7: *p* = 0.7757).

Thus, the data suggest a protective effect of netazepide on vincristine-induced mechanical allodynia. Netazepide 5 mg/kg restored normal mechanical sensitivity similar to Veh-Ctrl mice and significantly lower than Veh-VCR mice from D1 to D7 (D1: *p* = 0.0165, D5: *p* = 0.0023, D7: *p* = 0.0003 Veh-VCR vs. NTZ5-VCR) (Figure 1). Similar results were obtained with a lower dose of 2 mg/kg (D1: *p* = 0.0065, D3: *p* = 0.0005, D5: *p* = 0.0125, and D7: *p* = 0.0132 Veh-VCR vs. NTZ2-VCR).

### 2.2. Netazepide Alleviates the Decrease in IENF and DRG Neuron Densities Induced by Vincristine

**Figure 2:**
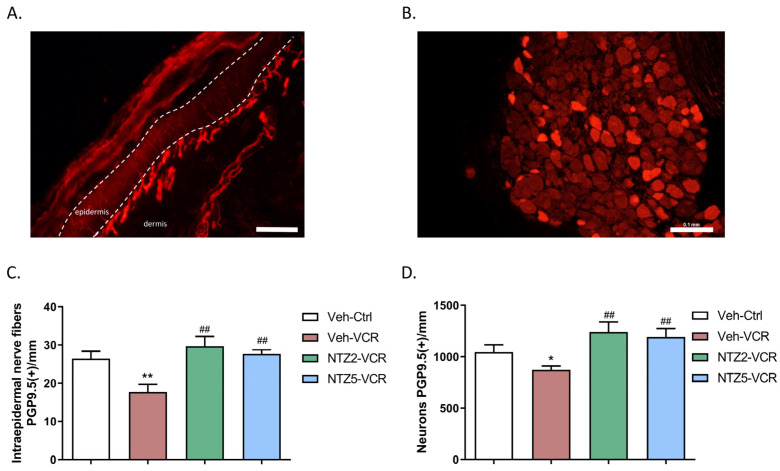
Effect of netazepide (2 or 5 mg/kg) on the loss of sensory nerve endings and neurons induced by vincristine in mice. Immunohistochemistry for PGP9.5 was performed on paw skin sections (**A**) and DRG (**B**). (**C**) Intraepidermal nerve fiber density was assessed. Three sections of paw skin were examined per mouse. n = 6 mice. (**D**) Quantification of DRG neuron density was evaluated. Three DRG sections and three DRG per mouse were counted. n = 6 per group. * *p* < 0.05, ** *p* < 0.01 vs. Veh-Ctrl; ## *p* < 0.05 vs. Veh-VCR. PGP9.5: protein gene product 9.5. Ctrl: control, NTZ2: netazepide at 2 mg/kg, NTZ5: netazepide at 5 mg/kg, Veh: vehicle, VCR: vincristine.

**Figure 3:**
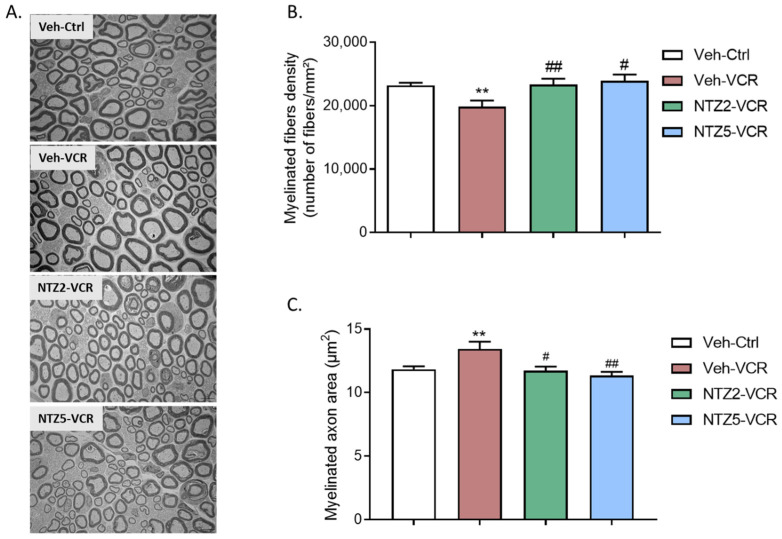
Quantitative analysis of sciatic nerves by electron microscopy. (**A**) Visualization of myelinated fibers in the sciatic nerve. (**B**) Quantification of myelinated fiber density. (**C**) Measurement of myelinated axon area in the nerve. n = 6 per group; ** *p* < 0.01 vs. Veh-Ctrl. # *p* < 0.05; ## *p* < 0.01; vs. Veh-VCR. Ctrl: control, Ly: Ly225910; NTZ2: netazepide at 2 mg/kg; NTZ5: netazepide at 5 mg/kg; Veh: vehicle; VCR: vincristine.

Netazepide at 2 or 5 mg/kg had no effect on IENF and DRG neuron densities in the Ctrl groups. The density of IENF was lower in Veh-VCR mice than in Veh-Ctrl mice (*p* = 0.0043). However, there was no significant difference in IENF density between Veh-Ctrl mice and Veh-VCR mice treated with netazepide at 2 or 5 mg/kg (Figure 2A). Similarly, the administration of vincristine resulted in a significant reduction in the density of DRG neurons. (*p* = 0.0325 Veh-Ctrl vs. Veh-VCR, Figure 3B). Treatment with netazepide prevented the vincristine-induced loss of DRG neurons (*p* = 0.0022, NTZ5-VCR vs. Veh-VCR, *p* = 0.0152, NTZ2-VCR vs. Veh-VCR) (Figure 2B).

### 2.3. Effect of Netazepide on Myelinated Nerve Fiber Density and Morphology in Sciatic Nerves on Vincristine-Induced Peripheral Neuropathy Model

Neither dose of netazepide had any noticeable effect on the morphology of myelinated or unmyelinated nerve fibers in the sciatic nerves of the control groups. However, there was no significant alteration in the morphology of unmyelinated fibers. (Figure 3A) in Veh-VCR mice relative to Veh-Ctrl mice. Quantitative analysis of electron microscopy images revealed a significant reduction in myelinated fiber density following vincristine treatment. (*p* = 0.0076, Figure 3B). This reduction was linked to an augmentation in the area of myelinated axons. (*p* < 0.01, Figure 3C). Netazepide prevented the decrease in myelinated fiber density at 2 or 5 mg/kg (*p* = 0.0206, Figure 3B) and the increase in myelinated axon area induced by vincristine (NTZ2-VCR: *p* = 0.0313, NTZ5-VCR: *p* = 0.0043 vs. Veh-VCR), such that there was no significant difference between the myelinated axon areas of mice in the Veh-Ctrl, VCR-NTZ2, and VCR-NTZ5 groups (Figure 3C).

## 3. Discussion

Overall, the main findings of this preclinical study are that CCK2R blockade by netazepide is effective in preventing the occurrence of painful symptoms, i.e., tactile allodynia, and sensory nerve injuries induced by repeated injection of vincristine in a mouse model.

Tactile allodynia is one of the predominant sensory symptoms in humans receiving vincristine. Mice exposed to vincristine also developed tactile allodynia from D1 to D7 after the start of vincristine injections, as found in other studies in rodent models [37,38,39]. An administration of netazepide in mice prevented the onset of vincristine-induced tactile allodynia at both tested doses. The lowest dose of 2 mg/kg was sufficient to prevent the onset of vincristine-induced pain. Allodynia is a complex process involving both peripheral and central mechanisms [40].

Among the pharmacological treatments that have been used to modulate neuropathic allodynia, drugs that act on opioid receptors have shown a beneficial effect. However, opioid administration is frequently associated with the onset of adverse effects, tolerance, and dependence [41]. One explanation for CCK2R blockade-induced analgesia is its capacity to dimerize with the opioid receptor MOR [42]. Indeed, upon activation, CCK2R forms heterodimers with MOR, preventing the binding of opioids to their receptor and so blocking their analgesic effect. Thus, CCK2R antagonists have been investigated in combination with opioids and proposed as morphine adjunct therapy [24,43,44,45]. Does YF476 (netazepide) cross the BBB? Following injection of carbon-11-labeled YF476 into the tail vein of rats, exceedingly low levels of radioactivity were found in all brain regions from 5 to 60 min post-injection [46]. Other investigators obtained similar results in rats [47,48]. However, Zhang et al. [49] showed that netazepide can cross the BBB and that neuropathic pain is maintained by brainstem neurons co-expressing opioid and cholecystokinin receptors (CCK2R-MOR) [50].

Vincristine does not cross the blood-brain barrier and is responsible for peripheral nerve injuries. Thus, we suppose that netazepide-induced analgesia in our vincristine-induced peripheral neuropathy model also involved peripheral mechanisms. Another explanation is that activation of CCK2R expressed in mouse DRG neurons could lead to neuronal hyperexcitability, resulting in pain hypersensitivity [51]. Actually, Yu et al., 2019 showed that activation of CCK2R by CCK-8 stimulated the Gα_o_ subunit, leading to the induction of a particular signal transduction pathway resulting in the inhibition of the *Ia* current in small-sized sensory neurons. A decrease in Ia, encoded by A-type K^+^ channels, caused an increase in excitability in small-sized sensory neurons [52]. Our functional results support the use of a CCK2R blocker alone as an efficient analgesic for the treatment of neuropathic pain.

Several studies have demonstrated that damage to small C and Aδ nociceptors and to low-threshold Aβ fibers leads to mechanical allodynia [40,53,54,55]. It is important to notice that a definitive diagnostic protocol is not present in the current literature, especially when the damage is to small fibers, and therapeutic strategies are lacking, especially for small fiber damage. This underscores the importance of developing new drugs [13,26,56].

These observations are in agreement with our histological findings. Immunofluorescence analysis of the skin demonstrates that vincristine induced damage to small nerve fibers is highlighted by a decrease in IENF density, as previously shown in rats [57]. Furthermore, electron microscopy analyses show that vincristine leads to a decrease in myelinated nerve fiber density and an enlargement of myelinated axons in the sciatic nerve, as previously shown [11,58,59]. Here, netazepide pretreatment at 2 and 5 mg/kg prevented vincristine-induced nerve injuries, suggesting a neuroprotective role for netazepide. Vincristine acts by inhibiting microtubule polymerization and mitotic spindle formation, stopping the cell cycle and leading to cancer cell death [60]. Integrity of the microtubule network is crucial for the peripheral neurons, although neurons do not divide. Microtubules maintain the elongated morphology of peripheral neurons, ensure axonal transport, and cause neuronal excitability [61]. Thus, structural alteration of myelinated nerve fibers exposed to vincristine could be associated with the microtubule-targeting agent activity of vincristine. However, other pathophysiological mechanisms are involved in vincristine-induced neurotoxicity, such as neuro-inflammation and mitochondrial dysfunction. Vincristine affects calcium movement by acting on the mitochondrial membrane [62]. This change in calcium flux alters mitochondrial function, resulting in a decrease in energy production and axon degeneration [63,64]. CCK2R belongs to the G protein-coupled receptor (GPCR) family and is preferentially associated with the G_αq_ protein, whose activation results in mobilization of intracellular Ca^2+^ and activation of Ca^2+^ dependent-signaling pathways [65]. Overstimulation by CCK-8 has been shown to induce mitochondrial changes in pancreatic acinar cells, leading to acute pancreatitis (González et al., 2003). Overexpression of CCK2R in DRG could alter calcium homeostasis, leading to mitochondrial dysfunction and energy loss in sensory neurons. Blocking CCK2R could limit intracellular Ca^2+^ accumulation and thus protect sensory neurons from degeneration. In addition, it was shown that activation of microglia and astrocytes in the spinal cord during exposure to vincristine leads to the release of cytokines, sustaining neuropathic pain through inflammatory mechanisms [38]. Blocking the CCK system may exert anti-inflammatory properties and prevent VCR-induced neuroinflammation. The overexpression of CCK2R mRNA in airway-innervating sensory neurons was observed after lung inflammation [66]. Upregulation of CCK2R in DRG in response to injury and/or inflammation may initiate either a neuroprotection/neuroregenerative process or an inflammatory process. Thus, the blockade of CCK2R may inhibit anarchic nerve sprouting, causing pain, or regulate an inflammatory mechanism leading to pain and neurodegeneration. An anti-inflammatory effect of CCK system blockade has already been demonstrated in a proglumide-treated model of chronic pancreatitis [67].

In our previous study, daily administration of proglumide accelerated the recovery of normal mechanical sensitivity in mice exposed to vincristine and prevented nerve injuries induced by vincristine [26]. Proglumide is a non-selective antagonist of CCK1R and CCK2R and has already been the subject of clinical trials. In the perspective of drug repositioning, netazepide showed more promising results, as its administration completely prevented the onset of mechanical allodynia induced by vincristine. However, the animal model used in this study is cancer-free. Thus, the effect of netazepide on tumor growth, the anticancer properties of vincristine, and the possible adverse effects associated with the interaction of both drugs could not be evaluated here. Further investigations are needed to determine the influence of netazepide on a tumor-bearing experimental model.

## 4. Materials and Methods

This study adhered to the ethical care guidelines for experimental animals of the European Community (Directive 2010/63/EU) and was submitted to the French Ministry of Higher Education and Research and approved (APAFiS # 27947-2020111216498126 v2). Animal experiments are presented in accordance with ARRIVE guidelines [68]. All efforts were made to minimize both suffering and the total number of animals employed in the experiments. A total of 60 male and female (30:30) Swiss mice (6–7 weeks old) from Janvier Labs (Saint Berthevin, France) were housed in groups of 4 to 5 per cage and kept under a 12 h light/dark cycle with food and water available ad libitum (BISCEm-animal care and facility center). Shredded paper nesting material was supplied for environmental enrichment. The mice were allocated to 3 groups: vehicle (Veh), netazepide at 2 mg/kg (NTZ2), and netazepide at 5 mg/kg (NTZ5). The mice in each group were further categorized into 2 subgroups: control (Ctrl) or vincristine (VCR), resulting in a total of 6 groups of mice (n = 10 in each group): Veh-Ctrl, NTZ2-Ctrl, NTZ5-Ctrl, Veh-VCR, NTZ2-VCR, and NTZ5-VCR. The number n was calculated according to the variability obtained during the von Frey test in previous studies using the same model [26,36].

The maximal dose of 5 mg/kg was calculated until the efficient dose to reduce the number of tumors in patients with multiple gastric neuroendocrine tumors (type 1 gastric NETs) Patients received 50 mg orally once a day [35]. Pharmacokinetics studies showed a bioavailability in non-clinical studies between 30 and 50% [69]. So, according to Nair and Jacob (2016), the equivalent dose for mice was: (50 mg × 0.4)/60 kg = 0.33 mg/kg for the human dose, which corresponds to 0.33 × 12.3 = 4.1 mg/kg for the mouse dose [70]. So, the dose of 5 mg/kg was defined as the maximal dose, and the dose of 2 mg/kg was defined as the minimal dose.

The assignment of mice to each group was conducted using an online randomization tool. (http://www.graphpad.com/quickcalcs/index.cfm (accessed on 28 September 2021)). Sciatic nerve, DRG, and paw skin were removed at the end of the experiment for immunohistochemistry and morphological analyses.

### 4.1. Treatments

**Figure 4:**
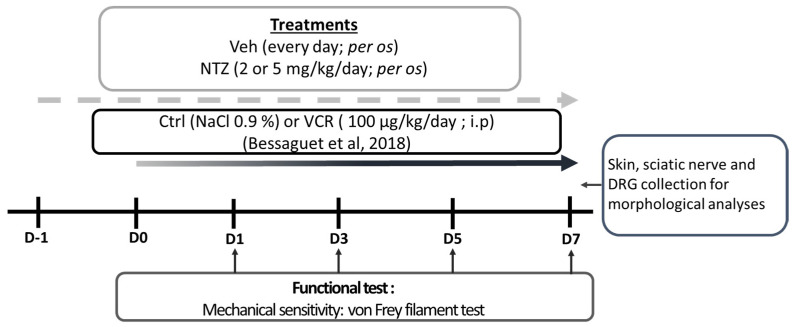
Schematic representation of the study design: Ctrl: control; DMSO: dimethyl sulfoxide; DRG: dorsal root ganglion; NTZ: netazepide; PEG 300: polyethylene glycol 300; Veh: vehicle [36].

Peripheral neuropathy was induced through daily intraperitoneal [i.p.], injections of vincristine at a dosage of 100 µg/kg for 8 consecutive days (Hospira, Meudon, France) [36], or an equivalent volume of the diluent (saline solution, i.p.) for Ctrl mice (Figure 4).

Treatments started one day before the first vincristine administration and were administered, per os, each following day for 8 days. Netazepide active pharmaceutical ingredient (API) 2 or 5 mg/kg was diluted in a final solution of 50% polyethylene glycol 300 (PEG 300) and 10% dimethyl sulfoxide diluted in saline solution (50% PEG 300, 40% NaCl 0.09%, 10% DMSO). Vehicle mice received an equivalent volume of 50% PEG 300, 40% NaCl 0.09%, and 10% DMSO.

### 4.2. Behavioral Test

A functional test was conducted on days 1, 3, 5, and 7 across all groups. Animals were acclimatized to the testing room for at least 1 h before behavioral testing. The behavioral test was assessed by the same researcher, blinded to the treatment (NTZ 2 mg/kg, NTZ 5 mg/kg, or Veh) and the conditions (VCR or Ctrl).

Tactile sensitivity was assessed using the von Frey filament test (Bioseb, France) [71]. Mice were placed in a plastic cage with a wire mesh floor, which allowed access to their paws for 30 min for acclimatization. The plastic cage was covered with an opaque cup to prevent visual stimulation. The testing focused on the mid-plantar left hind paw, and the mechanical threshold was assessed using a modified version of the simplified up-down method [72]. The test began with filament #6 (0.40 g) and advanced to higher or lower filament values based on the animal’s response. Each animal underwent three test rounds for each paw under each experimental condition.

### 4.3. Immunohistochemistry and Morphological Analyses

#### 4.3.1. Quantification of IENF and DRG Neuron Densities

To assess for sensory innervation, animals (n = 6 per group) were euthanized at D7 through cervical dislocation following isoflurane anesthesia.

Then, footpads were removed with a punch biopsy (diameter of 3 mm), fixed overnight at 4 °C in 4% paraformaldehyde (PFA 4%), cryoprotected overnight at 4 °C (sucrose 30%), and frozen at −80 °C. Sections of 20 µm were sliced using a cryostat and left to incubate overnight with the primary antibody directed against protein gene product 9.5 (PGP9.5, rabbit monoclonal, 1:50; Abcam, Cambridge, UK). Sections were then incubated with the secondary antibody Cy3-conjugated (1:500; Jackson Immunoresearch, Suffolk, UK). Epidermal nerve fibers were blindly counted under 400× magnification (Eclipse 50i, Nikon Instruments Inc., Melville, NY, USA), following established guidelines for humans [19]. The length of the dermo-epidermal junction was determined with NIS-Elements BR 2.30 software (Nikon Instruments Inc., Melville, NY, USA) and was defined as the epidermal length. Epidermal nerve density was defined as the number of epidermal nerves divided by the epidermal length. To assess the density of DRG neurons, four lumbar (L4–L5) DRG sections per mouse were collected and processed as described above, except that 8 µm sections were sampled. Each DRG section was photographed at 200× under a fluorescence microscope in a systematic fashion. Immunoreactive DRG neurons were counted, and only the area containing neurons was measured with NIS-Elements BR 2.30 software (Nikon Instruments Inc., Melville, NY, USA). The density of PGP 9.5(+) neurons was expressed as neurons/mm^2^.

#### 4.3.2. Sciatic Nerve Ultrastructural Analysis

To evaluate the morphology and quantification of myelinated nerve fibers, sciatic nerves were dissected and immersed in a 2.5% glutaraldehyde solution diluted in Sorensen buffer, dehydrated, and embedded in Epon 812 resin (Euromedex, Souffelweyersheim, France). Semi-thin sections were stained with toluidine blue. Ultrathin sections were stained with uranyl acetate and lead citrate. The observations were made using an electron microscope (JEM-1400 Flash, Jeol, Tokyo, Japan). Six photographs were captured per animal (n = 6 per group), covering the entire section of sciatic nerve, were taken at 3000× magnification, and the number of myelinated fibers per mm^2^ was counted to calculate a density.

### 4.4. Data Analysis

All data were presented as the mean +/− SEM. For multiple groups with a Gaussian distribution, a one-way analysis of variance (ANOVA) was employed to assess differences, and Tukey’s multiple comparisons test was utilized to determine *p* values. In cases where data did not follow a Gaussian distribution, a nonparametric Kruskal–Wallis test was conducted, and the Dunn multiple comparisons test was applied. Statistical significance was considered at *p* < 0.05.

## 5. Conclusions

These results provide evidence to support the concept that netazepide has analgesic and neuroprotective properties. Furthermore, CCK2R is expressed in various types of cancer, such as melanoma, pulmonary, and digestive cancers, and its antagonism alleviates cancer growth [73,74]. So, netazepide, a well-tolerated drug already investigated in clinical trials for other indications, is a potential good candidate in the context of vincristine-induced peripheral neuropathy because of its neuroprotective and anticancer properties.

## Data Availability

The data presented in this study are available on request from the corresponding author.
